# Are Saudis Equipped to Provide Adequate First Aid to Someone Having a Seizure?

**DOI:** 10.7759/cureus.24898

**Published:** 2022-05-11

**Authors:** Asma S Habbash, Khaled A Amer, Abdulrahman A Aldosari, Rammas A Shawkhan, Majdoleen A Abdulrahman, Shuruq Z Alshehri, Rahaf Y Wakidah

**Affiliations:** 1 Family Medicine, King Khalid University, Abha, SAU; 2 College of Medicine, King Khalid University, Abha, SAU

**Keywords:** saudi arabia, public, attitudes, first aid, epilepsy, knowledge

## Abstract

Background and aim

Epilepsy is one of the most common neurological disorders with a huge impact on the physical and psychological well-being of the individuals affected. Unwanted behavioral practices regarding epilepsy emergencies result in poor management, costly clinical interventions, and frequent unnecessary visits to the emergency departments. We aimed at conducting a large-scale investigation of behavioral practices, beliefs, and perceptions regarding epilepsy first aid measures among the Saudi public.

Methods

This is a descriptive questionnaire-based cross-sectional study of the general public residing in the Southwestern region of Aseer in Saudi Arabia.

Results

The study included 1230 participants. There were about 70.4% shows good knowledge about epilepsy and 74.2% with good knowledge about epilepsy first-aid. About 87.1% reported that they know what epilepsy really is, and nearly 38.1% know somebody diagnosed with epilepsy. Only 25.4 sought doctors for epilepsy-related knowledge. About 16.7% thought spirit possession to be the aetiology for epilepsy and about 14.1% believed that evil eye was the cause. In terms of the adjusted impact for background factors on epilepsy knowledge, high education, attending seminars, or getting information from doctors was associated with a better knowledge score. However, housewives were less knowledgeable in terms of epilepsy-related knowledge.

Conclusion

We uncovered in our present investigation the upward trend for behavioral practices regarding epilepsy during the last few years among the Saudi public. We noted that behavioral practices and knowledge levels about epilepsy were far better among highly educated graduates. One concerning finding is the huge reliance on media for information about epilepsy. The media may not be the ideal channel for the dissemination of health-wise information about epilepsy. Our study results showed that there was a very low level of first-hand experience of knowing some acquaintances living with epilepsy, likely because of stigma. Spirit possession and evil-eye beliefs are embedded in the Saudi culture. It was notable that 25% and 19% of minorities reported getting information about epilepsy from doctors and seminars, respectively. These were the two groups with the highest adjustable knowledge score though. Knowledge about first aid for epilepsy was satisfactory in several aspects. Sadly, housewives were less knowledgeable in terms of epilepsy-related knowledge than other categories of employment. Public healthcare facilities should be more proactive. Health education should be provided to the general public using simple and understandable language to help improve knowledge and attitudes towards epilepsy and all related chronic illnesses.

## Introduction

Epilepsy patients frequently attend the emergency room, which is expensive yet clinically unwarranted. It's possible that providing them and their caregivers with self-management intervention that enhances their confidence and capability to manage seizures will bring about fewer visits [1]. The neurological disorder epilepsy is very common - it affects one percent of the global population. Epilepsy is the most common neurological condition in children, with the majority of cases occurring in the first decade of life [2]. Epilepsy is a chronic disorder characterized by recurrent seizures, the most severe part of which is the inability to predict when and where the next seizure will occur. To combat the myths and misconceptions related to epilepsy, there is a definite need for rigorous health education for teachers on many facets of the disease [3]. Epilepsy affects 50 million individuals globally, with 40 million living in low- and middle-income areas. Children with epilepsy are more likely to have school underachievement, learning difficulties, mental health issues, social isolation, and low self-esteem than other children. The understanding of epilepsy by teachers and their attitudes to it can directly affect education, development of social skills, success after school in employment, social skills, and the development of social networks for schoolchildren with epilepsy [4]. Doctors have an important role in teaching patients, parents, and the general public how to respond to someone who has a seizure [5]. Despite having obtained prior counselling from a physician, parents of children with seizures usually forget what to do during seizure attacks [6]. Aside from the impairment caused by seizures or the adverse effects of antiepileptic drugs, the social stigma associated with epilepsy is sometimes a significant barrier for individuals who live with it [7]. Public perceptions of epilepsy, as well as public awareness and attitudes about it, can have a major influence on these issues, including an individual's performance, social skill development, and future employment [8]. Several emergency department visits by patients with epilepsy are clinically unnecessary following uncomplicated seizures that could be managed safely at home [8]. Almost one-third of adults responding to the 2002 Centers for Disease Control and Prevention Health Styles Survey the fundamental aspect that must be stressed in providing first aid for seizures is to protect the person experiencing the seizure from harm. We must have a plan to respond to repetitive or prolonged seizures and raise the awareness of first aid basics among caregivers and in the community [5]. Epilepsy may affect people of various ages, which is a common neurological illness. It is caused by abnormally high or synchronized neural activity [9]. Epilepsy is projected to afflict 50 million individuals globally, with 40 million of them living in low- and middle-income nations. People with epilepsy face social prejudice, bad attitudes, and stigma that is often more damaging and detrimental than the disease itself [4]. Patients with epilepsy are three times more likely to die young than the general population [9]. The current study aims at estimating the behavioral practices, beliefs, and perceptions regarding epilepsy and epilepsy-related first aid among the Saudi public residing in the Aseer region.

From December 2016 to May 2017, a population-based cross-sectional survey was undertaken in Tehran, Iran, which employed stratified cluster sampling at random. Data was gathered via interviews and a questionnaire. For the attitudes portion, which had 20 statements, the Likert scale was employed. The responses to the questions about first-aid measures were classed as either useful or harmful [10]. In total, 833 adults took part in the poll. Forty-one (4.9 %) participants had a very good total awareness score, 194 (23.3%) had a good score, 255 (30.6%) had a fair score, 210 (25.2%) had a low score, and 133 (16.0%) had a very low score. The mean (SD) score for general awareness was 4.6 (3.0), with a range of 0 to 11; causes were 5.8 (3.4), with a range of 0 to 13; seizure symptoms were 7.0 (4.0), with a range of 0 to 13; and first-aid measures were 7.5 (3.4), with a range of 0 to 14. At least one superstitious cause for epilepsy was mentioned by 260 (31.2%) of all participants. Except for marriage and having children, attitudes were typically positive [10]. A recent Kuwaiti study surveyed over 800 schoolteachers in terms of their knowledge about and attitudes towards epilepsy. It recommends that in an educator setting, information about epilepsy and seizure first aid be offered to increase teachers' perception of epilepsy as well as preparation to handle seizures. More teacher attitude research, as well as the resumed development and deployment of epilepsy education programs, are indeed required. To dispel preconceived ideas about epilepsy and break its stigmatization with it, learners and other professionals must collaborate to allay stereotypes about epilepsy and promote the development of positive attitudes among school instructors [11]. Alqahtani, in his cross-sectional study that aimed to investigate the knowledge of teachers in the Southern region of Saudi Arabia, included more than 300 teachers in the study, and all of them were male. The study shows that more than 70% of them had seen the epileptic attack, and they had a minimum knowledge regarding epilepsy, which needs to be improved through a structured program as epilepsy is prevalent in Saudi Arabia [12]. Thapa et al. conducted a study to assess the belief and knowledge of high-school students regarding epilepsy. The study included 1360 participants from 33 private schools, and shows that the student's knowledge regard epilepsy but the knowledge needs to be enhanced [13]. Kolahi et al. published a study which the objective to estimate the epilepsy first-aid knowledge among teachers in Iran. This cross-sectional study included teachers from 342 schools, and it shows inadequate knowledge and positive altitude regarding epilepsy [14]. Alkhotani et al. conducted a study in Makkah, Saudi Arabia to assess the teachers’ knowledge regarding seizure first aid. Out of 426 teachers, the majority, especially the females, showed insufficient knowledge regarding seizure first aid [15].

## Materials and methods

This study was a cross-sectional questionnaire-based descriptive study. The Research Ethics Committee at King Khalid University approved the study with approval number (ECM#2021-5806). The study included a random sample of the general public resident of the Aseer region, Saudi Arabia. All adults resident in the Aseer region, Saudi Arabia were included. Participants aged under 18 years were excluded. A cross-sectional study was carried out using a self-administered questionnaire formed of demographic, clinical characteristics, and questions related to epilepsy first aid behavioral practices, beliefs, perceptions, and knowledge level, the questionnaire was distributed through a different method by data collectors to ensure accuracy. The study included a random sample of public residents in the Aseer region, Saudi Arabia. 

Data analysis

Data was analyzed using the R Statistical Software version 3.4.1 (R Foundation, Indianapolis, USA). Categorical data (such as educational level and employment) were summarized using frequencies and displayed using tables and bar graphs. Numerical continuous data, such as the knowledge score, were summarized using means and standard deviations and displayed using box-and-whiskers plots. The adjusted effect of categorical variables on the outcome variable (knowledge score) was determined using multiple Poisson generalized linear regression modeling. The level of significance was set at p ≤ 0.05.

## Results

The total number of people approached to participate in the study was (n= 1250) in the Aseer region. There were (n = 1230) agreed to take part in the study (response rate = 98.4%). A detailed account of demographic results is shown in Table 1.

**Table 1:** Baseline demographics of the study participants P <0.05 (significant)

Factor	Count (n)	Percentage	Epilepsy knowledge	Statistical test	P-Value
Gender					
Male	703	57.2%	12.0	t = 0.0339	0.973
Female	527	42.8%	12.0		
Age					
Below 20	264	21.5%	11.9	F = 0.973	0.421
20-30	531	43.2%	12.2		
31-40	214	17.4%	11.8		
41-50	141	11.5%	11.9		
Above 50	80	6.5%	11.6		
Education					
Primary school	5	0.4%	10.2	F = 9.746	9.24X10^-8^
Secondary school	19	1.5%	11.4		
High school	234	19%	11.0		
Collage	875	71.1%	13.4		
High education	96	7.8%	12.1		
Employment					
Field job	215	17.5%	12.3	F = 10.95	4.26X10^-7^
Housewife	139	11.3%	10.7		
Office job	203	16.5%	11.5		
Other	673	54.7%	12.3		

In terms of epilepsy-related knowledge, we summed up the correct answers with a maximum potential score of 22 points. There were (n = 866, 70.4%) participants who scored over half of the questions correctly and were deemed of ‘good knowledge’ about epilepsy, in contrast to (n = 364, 29.6%) who were of ‘poor knowledge’. Notably, the mean epilepsy knowledge score was 12.0 points (SD = 3.4 points). Epilepsy knowledge scores ranged between 5 and 20 points. The median score was 12 points.

With regards to first aid knowledge, we also calculated a knowledge score. We found (n = 913, 74.2%) with good knowledge and (n = 317, 25.8%) with poor knowledge. The mean first-aid knowledge score was 9.9 points (SD = 3.3 points). First aid knowledge scores ranged between 3 and 17 points. The median score was 11 points.

**Figure 1:**
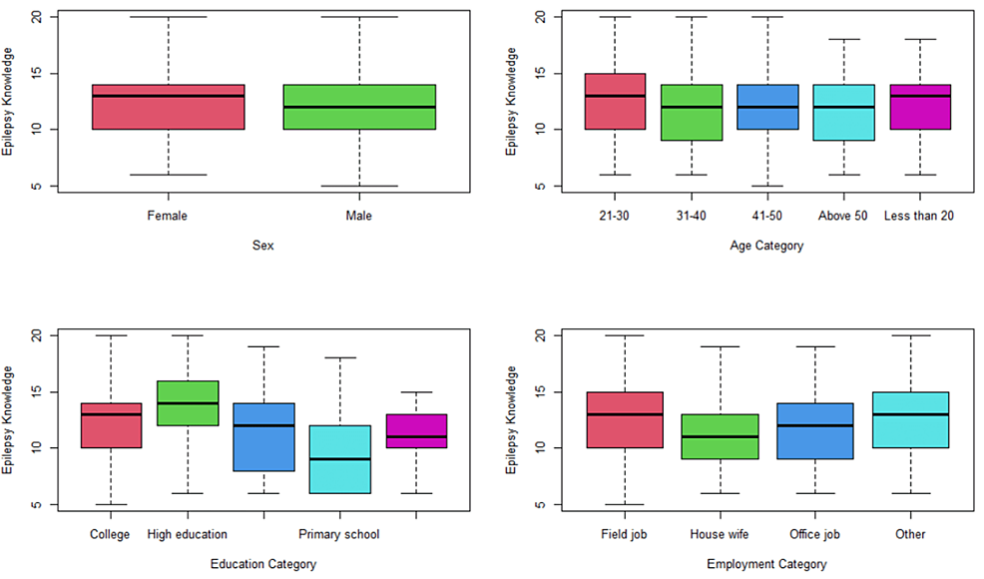
Epilepsy knowledge across different background categories

Males and females, as shown in Figure 1, were almost identical in terms of epilepsy knowledge (both scored a mean of 12 points). Those aged between 21 and 30 scored an average knowledge of 12.1 points, compared to the 11.6 points mean scored by the over-50s, however, the difference was not statistically significant (p = 0.421). The best knowledge score was found in Higher Education graduates (mean = 13.4 points), whereas the worst score was in Primary School graduates (mean = 10.2 points). This difference was statistically significant (p = 9.24X10-8). Among different employment categories, the mean epilepsy knowledge score ranged from 12.3 (among field workers) to 10.7 (among housewives). This difference was also statistically significant (p = 4.26X10-7).

**Figure 2:**
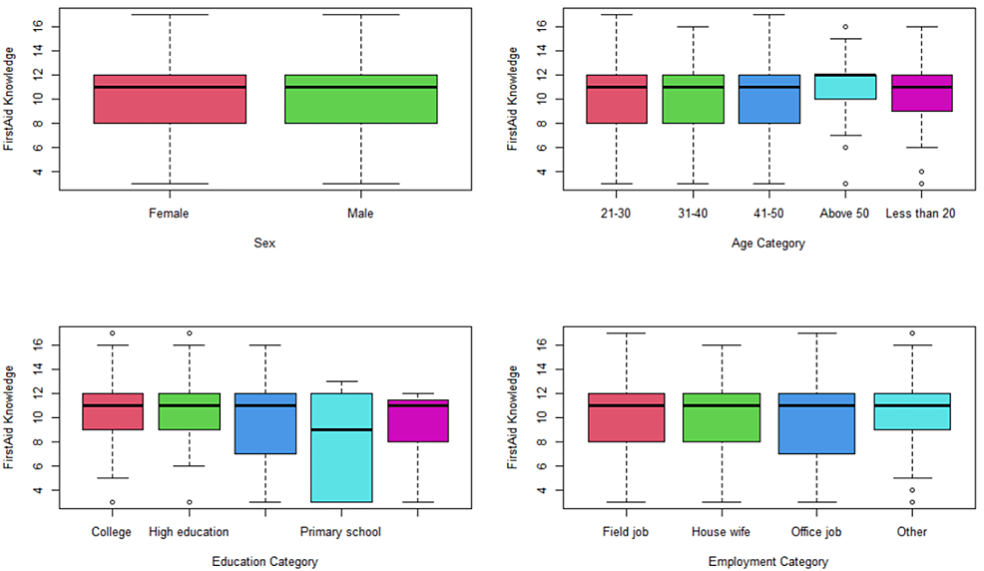
First aid knowledge across different background categories

Males and females, as on display in Figure 2, were no different in terms of first aid knowledge (both scored a mean of 9.9 points). Those aged above-50 scored the highest average knowledge score of 10.3 points, compared to the 9.6 points mean scored by the 31-40 years old, however, the difference was not statistically significant (p = 0.584). The best knowledge score was found in Higher Education graduates (mean = 10.3 points), whereas the worst score was in Primary School graduates (mean = 8.0 points). This difference was statistically significant (p = 0.0078). Among different employment categories, the mean first aid knowledge score ranged from 10 (among other workers) to 9.5 (among office job workers). This difference was not statistically significant (p = 0.074).

Among the participants, a majority (n = 1071, 87.1%) reported that they know what epilepsy is. Only (n = 469, 38.1%) of the participants knew somebody with an epilepsy diagnosis (Table 2). The most prevalent knowledge source was friends and relatives (n = 44.2%), followed by media (n = 469, 38.1%). Only (n = 312, 25.4%) were keen to seek doctors for epilepsy-related knowledge, with seminars only informing (n = 233, 18.9%) with their required knowledge. Some (n = 205, 16.7%) thought spirit possession to be the cause of epilepsy, and (n = 174, 14.1%) believed that evil eye was the cause.

**Table 2:** Epilepsy-related characteristics of the study participants

Factor	Count (n)	%
Do you know what epilepsy is?		
Yes	1071	87.1%
No	159	12.9%
Do you know personally someone with epilepsy?		
Yes	469	38.1%
No	761	61.9%
If the answer was "yes" how did you learn about it?		
Media	469	38.1%
Relatives or friends	544	44.2%
Seminars	233	18.9%
Doctors	312	25.4%
Other	186	15.1%
Do you know personally someone with epilepsy?		
Yes	540	43.9%
No	531	43.2%
What do you think is the cause of epilepsy?		
Genetic	556	45.2%
Tumour	195	15.9%
Infection	80	6.5%
Trauma	631	51.3%
Spirit possession	205	16.7%
Evil eye	174	14.1%
unknown	421	34.2%
What do you think are the symptoms of epilepsy?		
Loss of consciousness	786	63.9%
Falling	791	64.3%
Rolling of eyes	797	64.8%
Foaming of mouth	653	53.1%
Uncontrolled urination	786	63.9%
Biting of tongue	807	65.6%
Far gaze (strain)	340	27.6%
Choose the following statements that do you think is true:		
All children with epilepsy have the same symptoms.	67	5.4%
Epilepsy is confined to children only	10	0.8%
Epilepsy is a contagious disease	8	0.7%
Epilepsy Can be a cure	445	36.2%
None of the previous statements is true	611	49.7%
Do you think a child can have a seizure and not be recognized?		
Yes	888	72.2%
No	183	14.9%
Do you think epilepsy is a lifetime disorder?		
Yes	584	47.5%
No	487	39.6%
Do you know what to do if an epileptic child has an attack in front of you?		
Yes	497	40.4%
No	574	46.7%
What do you think is the most appropriate way to manage a child with epilepsy during the attack?		
Do nothing and call his parent	150	12.2%
Restrain the child	200	16.3%
Put something in his mouth to prevent his tongue from swallowing	785	63.8%
Keep him sitting or hold him upright	239	19.4%
None of the above	164	13.3%

The sheer majority (n = 1057, 85.9%) believed that seizure first aid is important, as can be seen in Table 3, with n = 1030, 83.7% emphasizing the lack of awareness about it. Some (n = 736, 59.8%) believed that it was wrong to restrain a person during a seizure attack.

**Table 3:** First aid perception-related characteristics of the study participants

Factor	Count (n)	Percentage
Do you think Seizure First Aid is Important?		
Yes	1057	85.9%
No	14	1.1%
Do you think society lacks awareness about the seizure of first Aid?		
Yes	1030	83.7%
No	41	3.3%
Do you think restraining the person during the attack is wrong behavior?		
Yes	736	59.8%
No	335	27.2%
If the answer was “Yes” chosen why?		
It may cause injury to me and themselves	741	60.2%
It’ll make the seizure get better	182	14.8%
How do you prevent a person during the attack from swallowing their tongue (You can choose more than one answer?		
Place a wallet or other	510	41.5%
Turn them on their side	577	46.9%
Hold their head still	315	25.6%
Do nothing	73	5.9%
After a seizure has passed and the person is fully awake, what should you do?		
Help the person sit in a safe place	638	51.9%
Tell them what happened in very simple terms	382	31.1%
Comfort the person and speak calmly	578	47%
Do you think Call 997 “the ambulance” is necessary?		
Yes	906	73.7%
No	165	13.4%
When do you think you need to call 997 “the ambulance”?		
If the seizure lasts longer than five minutes	959	78%
If the person is injured	901	73.3%
If the person has breathing difficulties after the jerking stops	988	80.3%
If it is the person’s first known seizure	812	66%

Most of the participants (n = 906, 73.7%) believed that a seizure necessitates calling the ambulance. Some (n = 988, 80.3%) thought breathing difficulties must be the main reason for involving emergency services, and further (n = 959, 78%) would do so with prolonged seizures, whereas (n = 901, 73.3%) would do it if a patient injured themselves and (n = 812, 66%) if it was the first-ever seizure for the individual involved.

In terms of the adjusted impact for background factors on epilepsy knowledge, high education, attending seminars, or getting information from doctors was associated with statistically significant higher knowledge score, as shown in Table 4. However, housewives were less knowledgeable in terms of epilepsy-related knowledge.

**Table 4:** The adjusted impact of participants’ characteristics on epilepsy-related perception SE: Standard error, p <0.05 (significant) *p < 0.05; **p < 0.01; ***p < 0.001

	Estimate	SE	Z-Value	P-Value
Gender: Male	-0.0072953	0.0173099	-0.421	0.67342
Age: 31-40	0.0123508	0.0279914	0.441	0.65904
Age: 41-50	-0.0110615	0.0309095	-0.358	0.72044
Age: Above 50	-0.0419269	0.0383644	-1.093	0.27446
Age: Less than 20	-0.0125712	0.0241706	-0.520	0.60299
Education: High education	0.0701656	0.0321822	2.180	0.02924 *
Education: High school	-0.0356703	0.0241899	-1.475	0.14032
Education: Primary school	0.1326220	0.1631487	0.813	0.41628
Education: Secondary school	-0.0434805	0.0716899	-0.607	0.54418
Employment: Housewife	-0.0956523	0.0354456	-2.699	0.00696 **
Employment: Office job	-0.0506391	0.0301328	-1.681	0.09285 .
Employment: Other	-0.0003271	0.0258950	-0.013	0.98992
Source: Media	0.0001767	0.0178148	0.010	0.99209
Source: Relatives	-0.0097406	0.0185855	-0.524	0.60021
Source: Seminars	0.0816060	0.0206917	3.944	8.02× 10^-05^ ***
Source: Doctors	0.0767826	0.0190798	4.024	5.72× 10^-05^ ***
Knew Someone with Epilepsy	0.0228711	0.0182924	1.250	0.21119

Table 5 shows that none of the background factors was impactful in terms of epilepsy first aid knowledge.

**Table 5:** The adjusted impact of participants’ characteristics on epilepsy first aid-related knowledge SE: Standard error, p <0.05 (significant)

	Estimate	SE	Z-Value	P-Value
Gender: Male	-8.422× 10^--03^	0.0188	-0.448	0.654
Age: 31-40	1.997× 10^--02^	0.0304	0.658	0.511
Age: 41-50	-4.209× 10^--03^	0.0335	-0.126	0.900
Age: Above 50	2.420× 10^--02 ^	0.0407	0.595	0.552
Age: Less than 20	-1.766× 10^-02^	0.0264	-0.670	0.503
Education: High education	-0.01142	0.0361	-0.317	0.752
Education: High school	-0.004394	0.0259	-0.170	0.865
Education: Primary school	0.02875	0.1746	0.165	0.869
Education: Secondary school	-0.09.878	0.0781	-1.265	0.206
Employment: Housewife	0.00959	0.0376	0.255	0.798
Employment: Office job	-0.01345	0.0329	-0.409	0.683
Employment: Other	0.01428	0.0285	0.501	0.617
Source: Media	0.004270	0.0194	0.221	0.825
Source: Relatives	6.971× 10^-05^	0.0203	0.003	0.997
Source: Seminars	0.01546	0.0230	0.673	0.501
Source: Doctors	0.3373	0.0210	1.608	0.108
Knew Someone with Epilepsy	0.02157	0.0200	1.081	0.280

## Discussion

The current investigation was carried out within the Aseer region and involved a large number of participants, over 1200, from the different sectors of the South-Western Saudi community. The response rate was an impressive 98.4% among the approached general public. The main result we uncovered in our current study is the 70.4% prevalence of good epilepsy-related knowledge and 74.2% prevalence of good epilepsy first aid-related knowledge among the general public in Southwestern Saudi Arabia. These results are far more impressive than the results found by Alhazzani et al. some six years earlier, in 2016, among the Saudi general public. A study conducted in Aseer, among 1000 general public participants, indicated a disappointingly low level of knowledge with regards to the nature of epilepsy, as many believed it was a hematological disease and, hence, a contagious disorder [16]. In 2018, a study from an Al-Kharj area in the central region of Saudi Arabia reported that nearly 70% of the general public were knowledgeable regarding the neurological nature of epilepsy (although nearly half the participants believed that epilepsy was related to evil eye-induced envy and/or demons’ possession) [17]. In 2019, in Al-Qaseem region [18], over two-thirds of the general public appreciated the fact that epilepsy is a brain disorder, a noticeable increase from the results found earlier [17]. That provides supportive evidence that knowledge about epilepsy may be improving during the last few years among the Saudi public, thanks to the technology-related information explosion. Notably, many countries could have a different experience in terms of decreasing knowledge levels over the last few years regarding epilepsy [19]. Although our finding is that three-quarters of participants showed a good level of knowledge, still many other countries have better knowledge about epilepsy than we do [20], making some room for improvement soon. Clearly, healthcare facilities should be more proactive. Health education should be provided to the general public using simple and understandable language to help improve knowledge and attitudes towards epilepsy and all related chronic illnesses. We noted that knowledge level about epilepsy was far better among highly educated graduates. Employment, age, and gender did not have any substantial impact on the general public in terms of epilepsy-related knowledge. Our results corroborate the findings found by [19]. The more educated and experienced would be more likely to have better epilepsy-related knowledge. One concerning finding is the huge reliance on media for information about epilepsy. That was noted even among recent surveys that showed most people revert to media and friends for information about epilepsy [21]. The media may not be the ideal channel for the dissemination of health-wise information about epilepsy [22]. Use of social media was also noted to be quite limited and of limited engagement. Researchers, for instance, have proposed prudent use of the so-called neuromarketing by media to help improve knowledge about epilepsy amongst other neurological disorders [22]. Many specialized groups, such as teachers, noted better use of healthcare professionals as a source of information rather than mainstream media [23]. Reassuringly, some evidence exists as to the minimum effect of negative portrayal of epilepsy in the media on epilepsy-related attitudes among college students [24]. Our survey results showed that there was a very low level of first-hand experience of knowing some acquaintances living with epilepsy. That was strange, although knowing someone with epilepsy had minimal impact on knowledge and attitudes [21], as the Saudi community is very well-connected. However, a huge stigma exists around epilepsy globally and in Saudi Arabia [25]. Therefore, families would make every effort to conceal such a diagnosis even from close relatives and friends.

Among our participants, there were nearly 17% believed spirit possession to cause epilepsy in contrast to 14% who thought that envy-related evil eye would be its cause. Such beliefs are embedded in the Saudi culture [26,27]. A recent systemic review reported levels as high as 25% for belief in possession and 35% for an evil eye as direct causes of epilepsy [9]. It was notable that 25% and 19% of minorities reported getting information about epilepsy from doctors and seminars, respectively. These were the two groups with the highest adjustable knowledge score though. One recent study showed that reliance on social media would improve the general knowledge about epilepsy but not the details [28] of, for instance, the etiology of epilepsy and appropriate management of epilepsy emergencies as highlighted in a recent systemic review [9]. Further surveys should seek to understand the reasons for the low uptake of information provided by doctors and seminars in Southwestern Saudi Arabia. One would speculate that such physician-led seminars made for the public would be scarce and, likely, non-existent. Over one-third of the Saudi public would consider preventing tongue biting by inserting something in a seizing patient's mouth [9]. Our current survey indicated that over 40% would do that. Certainly, plenty of important first-aid knowledge needs to be provided to lay people in Saudi Arabia. Their view is shared by over 83% of our surveyed participants. Nearly 60% of the people we investigated opposed restraining a seizing person. Knowledge about first aid for epilepsy was satisfactory in several aspects. Many knew the indication for calling emergency services during seizure attacks. However, nearly three-quarters were keen to engage ambulance services during epilepsy attacks. Sadly, housewives were less knowledgeable in terms of epilepsy-related knowledge than other categories of employment. This result is unique to our current survey. Public health services must reach out to housewives in terms of health education.

Study limitations and strengths

We note many strengths of the current study. Our research is the largest to be conducted among the general public in Saudi Arabia with over 1200 responses gathered and analyzed. One significant limitation in the current research would be the risk of social desirability bias that would have inflated the behavioral practices knowledge level, particularly towards first aid measures [29].

Future research should adopt an interventional design aiming at designing effective educational packages that could improve the behavioral practices and knowledge levels about epilepsy among the general public in Saudi Arabia. Preliminary evidence came up with promising results as to how such focused campaigns would raise awareness and improve health outcomes among the general Saudi public [30]. Further studies should seek to understand the reasons for the low uptake of information provided by doctors and seminars in Southwestern Saudi Arabia.

## Conclusions

In the current study, we uncovered in our present investigation the upward trend for behavioral practices regarding epilepsy during the last few years among the Saudi public. We noted that behavioral practices and knowledge levels about epilepsy were far better among highly educated graduates. One concerning finding is the huge reliance on media for information about epilepsy. The media may not be the ideal channel for the dissemination of health-wise information about epilepsy. Our study results showed that, likely because of stigma, there was a very low level of first-hand experience of knowing some acquaintances living with epilepsy. Spirit possession and evil-eye beliefs are embedded in the Saudi culture. It was notable that 25% and 19% of minorities reported getting information about epilepsy from doctors and seminars, respectively. These were the two groups with the highest adjustable knowledge score though. Knowledge about first aid for epilepsy was satisfactory in several aspects. Sadly, housewives were less knowledgeable in terms of epilepsy-related knowledge than other categories of employment. Public healthcare facilities should be more proactive. Health education should be provided to the general public using simple and understandable language to help improve knowledge and attitudes towards epilepsy and all related chronic illnesses. Physician-led seminars provided for the public would have the greatest impact on improving epilepsy-related knowledge. Such seminars should be encouraged, and the public should be invited to participate. Educational campaigns should not be hospital-based. Housewives are the least knowledgeable category in terms of epilepsy. Seminars and educational activities should reach out to them.

## References

[1] Noble AJ, Marson AG, Tudur-Smith C, Morgan M, Hughes DA, Goodacre S, Ridsdale L (2015). 'Seizure First Aid Training' for people with epilepsy who attend emergency departments, and their family and friends: study protocol for intervention development and a pilot randomised controlled trial. BMJ Open.

[2] Alkhamra H, Tannous A, Hadidi M, Alkhateeb J (2012). Knowledge and attitudes toward epilepsy among school teachers and counselors in Jordan. Epilepsy Behav.

[3] Goel S, Singh N, Lal V, Singh A (2014). Evaluating the impact of comprehensive epilepsy education programme for school teachers in Chandigarh city, India. Seizure.

[4] Eze CN, Ebuehi OM, Brigo F, Otte WM, Igwe SC (2015). Effect of health education on trainee teachers' knowledge, attitudes, and first aid management of epilepsy: an interventional study. Seizure.

[5] O'Hara KA (2007). First aid for seizures: the importance of education and appropriate response. J Child Neurol.

[6] Xiang XM, Miller D (2020). Effects of simulation video on parental recall of seizure first aid: a quality improvement project. J Child Neurol.

[7] Owolabi LF, Shehu NM, Owolabi SD (2014). Epilepsy and education in developing countries: a survey of school teachers' knowledge about epilepsy and their attitude towards students with epilepsy in Northwestern Nigeria. Pan Afr Med J.

[8] Abulhamail AS, Al-Sulami FE, Alnouri MA, Mahrous NM, Joharji DG, Albogami MM, Jan MM (2014). Primary school teacher's knowledge and attitudes toward children with epilepsy. Seizure.

[9] AlHarbi FA, Alomari MS, Ghaddaf AA, Abdulhamid AS, Alsharef JF, Makkawi S (2021). Public awareness and attitudes toward epilepsy in Saudi Arabia: a systematic review and meta-analysis. Epilepsy Behav.

[10] AB AB M, KO AA, FA AR, KE S (2019). Public awareness, attitudes, and first-aid measures on epilepsy in Tehran. Iran J Child Neurol.

[11] Al-Hashemi E, Ashkanani A, Al-Qattan H (2016). Knowledge about epilepsy and attitudes toward students with epilepsy among middle and high school teachers in Kuwait. Int J Pediatr.

[12] Alqahtani JM (2015). Knowledge and practice of schoolteachers towards students with epilepsy in Khamis Mushate, Southern Saudi Arabia. J Family Community Med.

[13] Thapa L, Bhandari TR, Shrestha S, Poudel RS (2017). Knowledge, beliefs, and practices on epilepsy among high school students of Central Nepal. Epilepsy Res Treat.

[14] Kolahi AA, Ghorbanpur-Valukolaei M, Abbasi-Kangevari M, Farsar AR (2018). Knowledge, attitudes, and first-aid measures about epilepsy among primary school teachers in northern Iran. Acta Neurol Scand.

[15] Alkhotani AM, Almalki WM, Alkhotani AM, Turkistani MA (2019). Makkah female teachers' knowledge of seizure first aid. Epilepsy Behav.

[16] Alhazzani AA, Alqahtani AM, Abouelyazid A (2016). Public awareness, knowledge, and attitudes toward epilepsy in the Aseer region, Saudi Arabia - a community-based cross-sectional study. Epilepsy Behav.

[17] Al-Dossari KK, Al-Ghamdi S, Al-Zahrani J (2018). Public knowledge awareness and attitudes toward epilepsy in Al-Kharj Governorate Saudi Arabia. J Family Med Prim Care.

[18] Alsohibani A, Alkheder R, Alharbi M, Alrasheedi M, Alsoghair M, Alsuhaibani M (2019). Public awareness, knowledge, and attitudes regarding epilepsy in the Qassim region, Saudi Arabia. Epilepsy Behav.

[19] Nagamori C, Hara K, Hirose Y, Ohta K, Akaza M, Sumi Y (2018). Public awareness and experiences associated with epilepsy in Japan, 2013-2017. Epilepsy Behav.

[20] Vodopić S, Vujisić S (2017). Public awareness, understanding and attitudes towards epilepsy in Montenegro. Acta Clin Croat.

[21] Kobau R, Zack MM (2021). Knowledge of and familiarity with epilepsy in U.S. adults: results from the 2017 ConsumerStyles Online Panel Survey. Epilepsy Behav.

[22] Javor A, Koller M, Lee N, Chamberlain L, Ransmayr G (2013). Neuromarketing and consumer neuroscience: contributions to neurology. BMC Neurol.

[23] Mott J, Shellhaas RA, Joshi SM (2013). Knowledge of epilepsy and preferred sources of information among elementary school teachers. J Child Neurol.

[24] Okumura A, Nakazawa M, Abe S, Ikeno M, Igarashi A, Shimizu T (2015). Sustained improvement of attitudes about epilepsy following a reduction in media coverage of car accidents involving persons with epilepsy. Epilepsy Behav.

[25] Tayeb HO (2019). Epilepsy stigma in Saudi Arabia: the roles of mind-body dualism, supernatural beliefs, and religiosity. Epilepsy Behav.

[26] Al-Habeeb TA (2003). A pilot study of faith healers’ views on evil eye, jinn possession, and magic in the Kingdom of Saudi Arabia. J Family Community Med.

[27] Obeid T, Abulaban A, Al-Ghatani F, Al-Malki AR, Al-Ghamdi A (2012). Possession by 'Jinn' as a cause of epilepsy (Saraa): a study from Saudi Arabia. Seizure.

[28] Alzhrani SH, AlSufyani MH, Abdullah RI, Almalki S (2021). Schoolteacher's knowledge, attitudes, and practice toward student with epilepsy in Taif, Saudi Arabia: cross-sectional study. J Family Med Prim Care.

[29] Del Brutto OH, Mera RM (2016). The importance of people compliance (social desirability bias) in the assessment of epilepsy prevalence in rural areas of developing countries. Results of the Atahualpa Project. Epilepsia.

[30] Alaqeel A, Kamalmaz H, Abou Al-Shaar H (2015). Evaluating the initial impact of the Riyadh Epilepsy Awareness Campaign. Epilepsy Behav.

